# Caseous calcification of the mitral annulus with mitral regurgitation and impairment of functional capacity: a case report

**DOI:** 10.1186/1752-1947-2-205

**Published:** 2008-06-12

**Authors:** Giovanni Minardi, Carla Manzara, Giovanni Pulignano, Paolo G Pino, Herribert Pavaci, Martina Sordi, Francesco Musumeci

**Affiliations:** 1 Department of Cardiology and Cardiovascular Surgery, Azienda Ospedaliera San Camillo-Forlanini, Rome, Italy; 2 Second Division of Cardiology, Department of Heart and Great Vessels, Attilio Reale, Sapienza, University of Rome, Italy

## Abstract

**Introduction:**

Mitral annular calcification is a common echocardiographic finding, especially in the elderly. Caseous calcification of the mitral annulus, however, is a relatively rare variant, having an echocardiographic prevalence of 0.6% in patients with mitral annular calcification. Caseous calcification needs to be differentiated from infected mitral annular calcification, mitral annular abscess and tumours. It is not malignant, and medical therapy with clinical follow-up is the therapeutic option. Surgery should be reserved for co-existent mitral valve dysfunction.

**Case presentation:**

We report the case of a 69-year-old woman, in whom caseous calcification of the mitral annulus was found at transthoracic echocardiography. Cardiac surgery was performed because of significant mitral regurgitation and impairment of functional capacity.

**Conclusion:**

Caseous calcification of the mitral annulus needs to be considered and confirmed by transthoracic echocardiography since there is potential for diagnostic confusion or misdiagnosis. This lesion appears to have a benign prognosis but, when associated with mitral valve dysfunction, cardiac surgery appears to be the best therapeutic option.

## Introduction

Mitral annular calcification (MAC) is a chronic degenerative process, which occurs mainly in older patients, particularly in women and in patients with end-stage renal failure on chronic dialysis [1]. Caseous calcification of the mitral annulus (CCMA) is a relatively rare variant with an echocardiographic prevalence of 0.6% in patients with MAC and 0.06% to 0.07% in large series of patients of all ages [2,3].

We describe a patient who was referred to our echocardiographic laboratory because of progressive impairment of functional capacity (up to New York Heart Association (NYHA) class III), and in whom moderate to severe mitral regurgitation (MR) and CCMA were found.

## Case presentation

A symptomatic 69-year-old woman (NYHA functional class III) underwent a transthoracic echocardiographic (TTE) examination to assess her left ventricular function. Her past history included hypercholesterolaemia, hypothyroidism and paroxystic atrial fibrillation. A DDD type pacemaker had been implanted due to sick sinus syndrome one year previously. She had marked limitation of physical activity. She was comfortable at rest but breathless on mild exertion. Physical examination revealed a pansystolic murmur of grade 3/6 audible in the mitral area. An electrocardiogram was completely normal. Laboratory examinations were as follows: haemoglobin 12.3 g/dl, glycaemia 72 mg/dl, urea 37 mg/dl, creatinine 0.9 mg/dl, calcium 8.7 mmol/l, phosphate 3.4 mmol/l, serum cholesterol 217 mg/dl and tryglicerides 148 mg/dl.

**Figure 1:**
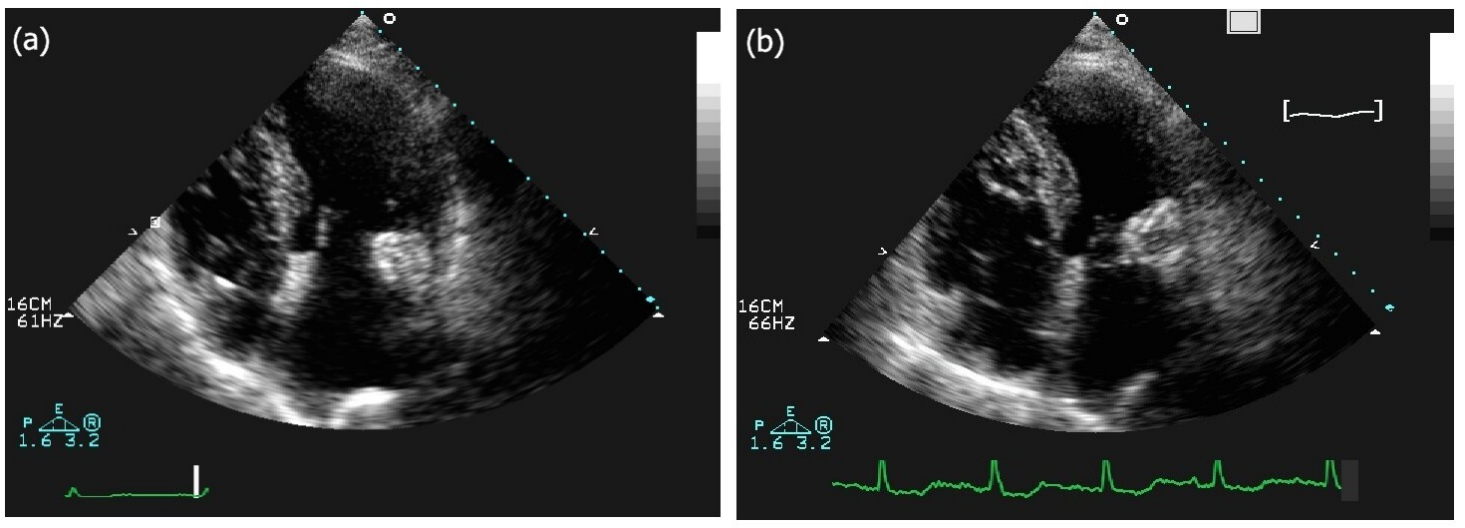
**Two-dimensional echocardiogram, apical four-chamber view**. (a) Pre-operative: a round echodense large mass attached to the calcified mitral annulus is seen. (b) Postoperative: a smaller round echodense mass attached to the calcified annulus is seen.

**Figure 2:**
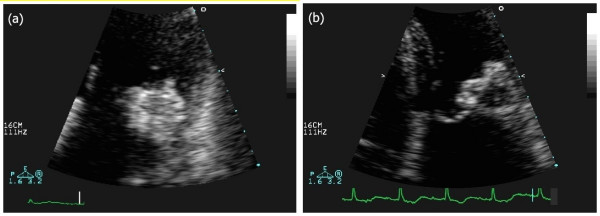
**Two-dimensional echocardiogram, apical four-chamber view (detail)**. (a) Pre-operative: dyshomogeneous echodensity of the mass is evident. (b) Postoperative: the mass has central echolucency surrounded by a hyperechogenic region.

**Figure 3:**
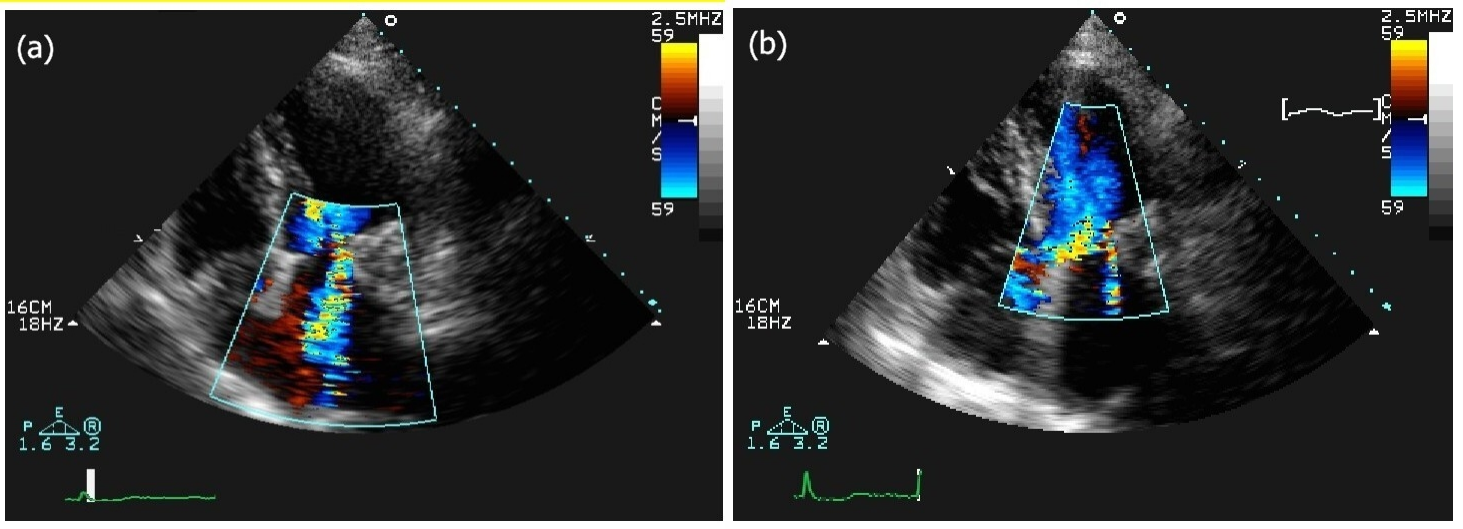
**Two-dimensional echocardiogram, apical four-chamber view, colour Doppler**. (a) Pre-operative: moderate to severe mitral regurgitation is present. (b) Postoperative: trivial mitral regurgitation is seen in the left atrium.

TTE revealed an echodense spherical, tumour-like mass (3.0 × 3.5 cm) located in the peri-annular posterior region close to the atrial side of the posterior mitral leaflet with an internal echolucent area, without acoustic shadowing (Figures 1a and 2a). On Doppler colour flow mapping, moderate to severe mitral regurgitation was seen in the left atrium, but no obstruction to the diastolic transmitral flow was found (Figure 3a). The left ventricle was hypertrophic (interventricular septum at end of diastole was 16 mm, left ventricular posterior wall at end of diastole was 14 mm) without wall motion abnormalities. The left atrium was dilated (anteroposterior diameter 48 mm). The right ventricle and right atrium were normal and the pacemaker lead was confirmed as inserted normally. The aortic valve was tricuspid and showed some calcification with mild stenosis and regurgitation.

Transoesophageal echocardiography (TEE) was performed to better evaluate the mass. TEE confirmed the previous findings, contributing more precise and detailed imaging regarding the internal echolucent area and showing the absence of systolic flow in the cavity. A multislice computed tomography (CT) scan of the heart was also performed, and the presence of a calcified round mass corresponding to the mitral valve was confirmed. The patient, being over 50 years old, underwent coronary angiography to exclude coronary artery disease, and then underwent cardiac surgery.

At surgery, the nodular mass was lanced with a longitudinal section along the mitral annulus for all its length, and the caseous white material that filled the centre of the mass was drained. Posterior ring annuloplasty with a GORE-TEX^® ^tube was performed with the aim of improving valve competence.

Repeat TTE, performed 7 days after surgery, showed a smaller round mass with calcified walls and a smaller internal anechogenic area as a result of the drainage (Figures 1b and 2b). Trivial mitral regurgitation was seen (Figure 3b). The patient was discharged after seven days with symptomatic improvement and was sent to a rehabilitation facility. Further follow-up study will be necessary as periodic assessment is important in this condition.

## Discussion

MAC is a common echocardiographic finding, especially in the elderly [4-6]. CCMA is a rare and relatively unknown aspect of MAC whose pathogenetic mechanism has not yet been defined [7]. It is easily recognized on M-mode and two-dimensional echocardiography as a round mass with a central echolucent area composed of a putty-like admixture of fatty acids, cholesterol and calcium. Due to its unusual characteristics, it may be misdiagnosed as a tumour or myocardial abscess, leading in some cases to unnecessary cardiac surgery [2,5,6]. The distinction between CCMA and a tumour should be based on the different clinical presentations. The typical location of calcification, the possible extension to the whole mitral annulus, sometimes involving the base of both mitral leaflets and/or to papillary muscles and chordae tendinae are characteristic of CCMA, as are the well-defined borders, the internal echolucent area and/or the possible acoustic shadowing, if a high degree of calcific deposit is present. In some cases TEE can add more precise information regarding the internal area of the mass, and cardiac fast CT, magnetic resonance imaging or single photon emission CT could also be useful. However, in some cases an intramyocardial tumour cannot be ruled out completely on imaging studies.

The distinction between CCMA and mitral annulus abscess should be based on their different clinical presentations. The lack of a large amount of calcification and its location at the mitral-aortic fibrosa, sometimes with systolic flow in the cavity visualized by colour Doppler, are characteristics of a mitral abscess.

CCMA is not a malignant disease. Surgery should be reserved for cases of uncertain diagnosis [5,6] and/or because of co-existent mitral valve dysfunction. In the latter situation it can be hypothesized that massive calcification may modify mitral annular dynamics and compromise the mitral leaflets' coaptation sufficiently to cause valvular regurgitation [7].

We have previously observed another case of CCMA: an asymptomatic 84-year-old woman who underwent TTE to assess cardiac function before vascular surgery. The patient had been on haemodialysis for 8 years because of chronic renal failure caused by both hypertension and diabetes mellitus. There was no associated mitral valvular dysfunction and left ventricular function was good; therefore, the chosen treatment was conservative, as CCMA is capable of spontaneous resolution, as reported previously [7]. In this patient we scheduled clinical and TTE follow-up yearly.

The noteworthiness of the case reported in this manuscript is that the patient had impairment of functional capacity (NYHA III functional class) and CCMA was responsible for moderate to severe mitral regurgitation. Cardiac surgery with mitral ring annuloplasty was the best option to improve cardiac haemodynamics and reduce the patient's symptoms.

## Conclusion

CCMA is a rare form of peri-annular calcification that needs to be considered and confirmed using TTE since otherwise there is a risk of diagnostic confusion or misdiagnosis. Once correctly identified with TTE the patient should be treated with medical therapy and clinical follow-up unless it is associated with mitral valve dysfunction, when cardiac surgery appears to be the best therapeutic option. Regular clinical and echocardiographic follow-up is recommended.

## Abbreviations

CCMA: caseous calcification of the mitral annulus; CT: computed tomography; MAC: mitral annular calcification; MR; mitral regurgitation; NYHA: New York Heart Association; TEE: transoesophageal echocardiography; TTE: transthoracic echocardiography.

## Competing interests

The authors declare that they have no competing interests.

## Authors' contributions

GM and PGP made substantial contributions to the conception and design of the study. GM, CM, GP and PGP made substantial contributions in the acquisition of clinical and echocardiographic data, GM, HP and MS were involved in drafting the manuscript and revising it critically, FM performed cardiac surgery, GM gave final approval of the version to be published. All authors read and approved the final manuscript.

## Consent

Written informed consent was obtained from the patient for publication of this case report and any accompanying images. A copy of the written consent is available for review by the Editor-in-Chief of this journal.
